# An In-Vitro Evaluation of Articulation Accuracy for Digitally Milled Models vs. Conventional Gypsum Casts

**DOI:** 10.3390/dj10010011

**Published:** 2022-01-11

**Authors:** Jason D. Lee, German O. Gallucci, Sang J. Lee

**Affiliations:** Department of Restorative Dentistry and Biomaterials Science, Harvard School of Dental Medicine, Boston, MA 02115, USA; german_gallucci@hsdm.harvard.edu (G.O.G.); Sang_Lee@hsdm.harvard.edu (S.J.L.)

**Keywords:** intraoral scanner, digital articulation, milled model, T-scan

## Abstract

With the advent of a digital workflow in dentistry, the inter-occlusal articulation of digital models is now possible through various means. The Cadent iTero intraoral scanner uses a buccal scan in maximum intercuspation to record the maxillomandibular relationship. This in-vitro study compares the occlusion derived from conventionally articulated stone casts versus that of digitally articulated quadrant milled models. Thirty sets of stone casts poured from full arch polyvinyl siloxane impressions (Group A) and thirty sets of polyurethane quadrant models milled from digital impressions (Group B) were used for this study. The full arch stone casts were hand-articulated and mounted on semi-adjustable articulators, while the digitally derived models were pre-mounted from the milling center based on the data obtained from the buccal scanning procedure. A T-scan sensor was used to obtain a bite registration from each set of models in both groups. The T-scan data derived from groups A and B were compared to that from the master model to evaluate the reproducibility of the occlusion in the two groups. A statistically significant difference of the contact region surface area was found on #11 of the digitally articulated models compared to the master. An analysis of the force distribution also showed a tendency for a heavier distribution on the more anterior #11 tooth for the digitally articulated models. Within the limitations of this study, the use of a digitally articulated quadrant model system may result in a loss of accuracy, in terms of occlusion, the further anteriorly the tooth to be restored is located. Care must be taken to consider the sources of inaccuracies in the digital workflow to minimize them for a more efficient and effective restorative process.

## 1. Introduction

Multiple techniques and materials have been used for the articulation of conventional dental models in their desired maxillomandibular relationship. Commonly, a recording medium such as wax or polyvinylsiloxane is used to transfer the patient’s maxillomandibular relationship to the casts for mounting. However, it is not always possible to achieve the desired accuracy clinically. Possible reasons for this include the dimensional instability of the recording material as well as the inability to fully seat the record due to inaccurate casts. Multiple studies have shown both vertical and horizontal discrepancies with conventional recording materials. These materials can undergo dimensional distortion under different temperature and moisture conditions [1,2,3], as well as over time [3,4,5,6]. Due to the dimensional instability of interocclusal registration materials, when adequate stable occlusal stops are present, the hand articulation of the casts has been shown to be a more accurate means of locating the proper interocclusal relationship [7,8]. The dimensional stability and accuracy of dental casts has been studied by multiple authors but can vary greatly depending on factors such as the impression and stone material used, impression technique, and storage time [9,10].

The popularity of computer-aided design and computer-aided manufacturing (CAD-CAM) generated restorations is on the rise as the technology and materials continue to evolve. Recent studies on these types of restorations have shown comparable clinical outcomes, and the digital workflow was proven to be the more efficient and the preferred method by both clinicians and patients [11,12,13,14,15]. The outcome of the restorations fabricated by CAD-CAM is dependent on the accuracy of the scan, casts, articulation and fabrication process through milling or 3D printing. Several studies have shown that the digitally fabricated casts and digital articulation process utilizing buccal scans are comparable to or more accurate than conventional methods [16,17,18]. However, there is a lack of information regarding the accuracy of the occlusal relationship derived from the digital articulation of digital models, specifically quadrant scans and models.

In order to assess the accuracy of the articulation of two mounted casts, a precise measurement of the occlusal contacts and their corresponding forces is necessary. Various methods for recording areas of occlusal contact have historically been used, including articulating foils, paper, waxes, silicone impressions, and photocclusion [3,19]. However, barring complex photoanalytical processes, most of these methods leave the analysis of the markings up to a subjective operator interpretation rather than outputting a quantifiable value. The T-scan III is a digital occlusal analysis instrument which uses a pressure sensitive sensor to allow the simultaneous registration and imaging of the relative distribution of occlusal forces and the contact time sequence. Furthermore, the results produce an immediate output of quantifiable data values as percentages of the load registered by the sensor as opposed to occlusal markings that must be qualitatively interpreted. Previous studies have shown contradictory results regarding the accuracy and reproducibility of previous generations of this system, with some showing an excessive variability in readings and a questionable reliability [20,21]. This may be attributable to the variable positioning of the digital pressure sensor as well as the sensitivity of the sensor. Others have found a very high level of precision [22,23,24], but recent literature has shown the T-scan III to have an improved precision and reliability when it comes to the measurement of the relative force distribution and number of contacts [25,26]. In this study, the digital occlusal markings from the digital pressure sensor were filtered through an imaging software in order to eliminate the artifacts from its sensitivity and precisely isolate the occlusal contacts. 

The aim of our in-vitro study was to determine the accuracy of the digital articulation of milled polyurethane quadrant models using a buccal scan versus that of conventionally mounted stone casts using a digital occlusal analysis device. The null hypothesis is that there will be no difference in the accuracy of the occlusion of digitally articulated milled models and conventionally articulated stone models.

## 2. Materials and Methods

### 2.1. Study Design

Mounted custom typodont master models were both conventionally impressed and digitally scanned by 30 different participants at the Harvard School of Dental Medicine. The resultant 30 sets of stone casts poured in type III stone (Microstone, Whip Mix Corp, Louisville, KY, USA) from full arch polyvinyl siloxane impressions (Aquasil, Dentsply Sirona, Waltham, MA, USA) (Group A) and thirty sets of polyurethane quadrant models milled from digital impressions (Itero Cadent) (Group B) were used for this study. 

**Figure 1:**
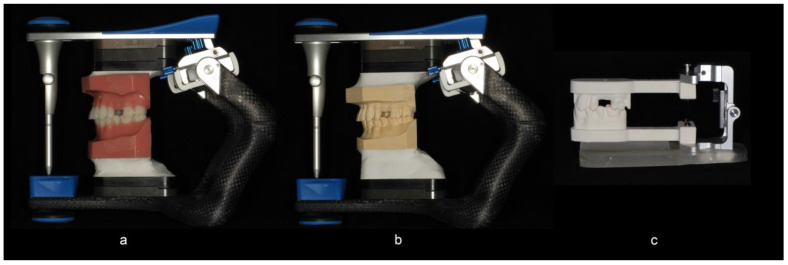
(**a**) The master model and (**b**) conventional stone casts were mounted on a semi-adjustable articulator. (**c**) The milled quadrant models were mounted on a prefabricated articulator (Itero Cadent Articulator) from the milling center based on the data obtained from the buccal scanning procedure.

**Figure 2:**
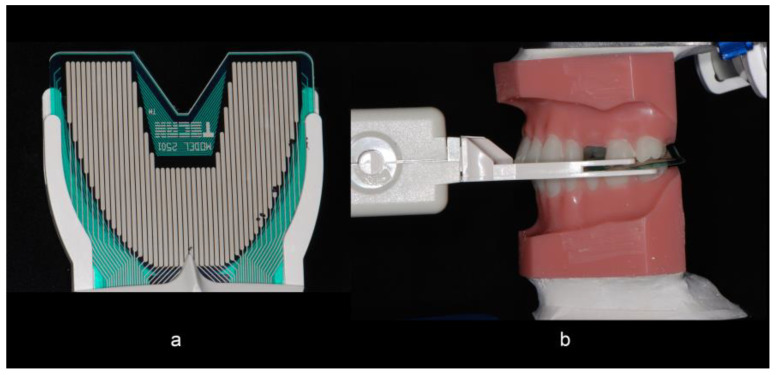
(**a**) The T-scan sensor was positioned on a fixed positioning jig to obtain a repeatable position for taking the occlusal reading. A weight of 1.5 lbs was placed over (**b**) the upper member of the articulator for each pair of models in groups A and B to obtain the reading.

In Group A, the full arch stone casts were hand-articulated and mounted on semi-adjustable articulators (Artex CR, Amann Girrbach AG, Pforzheim, Germany) with a low expansion mounting stone (Mounting stone, Whip Mix Corp, Louisville, KY, USA) uniformly in relation to the hinge-axis by the use of a mounting jig (Figure 1a–c). In Group B, the digitally derived quadrant models were mounted on a prefabricated articulator (Itero Cadent Articulator, Itero, Carlstadt, NJ, USA) from the milling center based on the data obtained from the buccal scanning procedure. A T-scan (T Scan III, Tekscan Inc., Boston, MA, USA) bite registration was obtained at a uniform position on the T-scan sensor with a weight of 1.5 lbs placed over the upper member of the articulator (Figure 2a,b) for the master model and for each pair of models in Group A and Group B. The same sensor was used between the paired groups. The relative occlusal pressures for teeth #11, 12, and 14 within each group were recorded directly from a digital occlusal analysis device. The subsequent force distribution values for each tooth were converted to a percentage of the sum of pressures on the three teeth.

**Figure 3:**
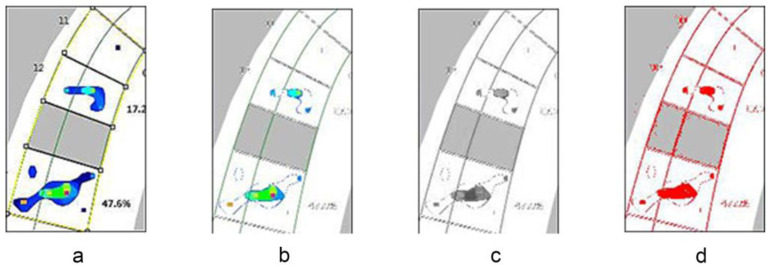
The T-scan graphical readouts were processed in a (**a**–**d**) step-wise process and analyzed in an image analysis software to eliminate artifacts from the readings.

The graphical T-scan readouts were transferred to an image analysis software (ImageJ; NIH, Bethesda, MD, USA). This software allows for a uniform reading of the exact areas corresponding to each tooth on the T-scan readout and allows for the elimination of artifacts from these readings. Based on discussions with a T-scan expert, it was determined that any regions of the readouts displaying a dark blue color should be considered artifacts and were thus eliminated from the analysis using the software (Figure 3a–d). The areas of the T-Scan readout corresponding to each tooth in question were individually cropped so that the same exact number of pixels was analyzed within each tooth group for every set of readings. The surface areas (pixels) of the remaining contact regions were then calculated.

### 2.2. Statistical Analysis

A sample size of thirty was used as this was an initial pilot study in this subject and thirty models were adequate for using a nonparametric test if the results were not normally distributed. A normality test was conducted prior to using the Kruskal Wallis nonparametric comparison. Following the Kruskal Wallis test, a post-hoc Wilcoxon rank sum test and a Bonferroni correction were used to adjust for multiple comparisons using a statistical software program (IBM SPSS Statistics, v22; IBM Corp, Armonk, NY, USA). The multiple comparisons were performed by using the Bonferroni method. Descriptive statistics were used to present a percentage force distribution by tooth.

## 3. Results

**Figure 4:**
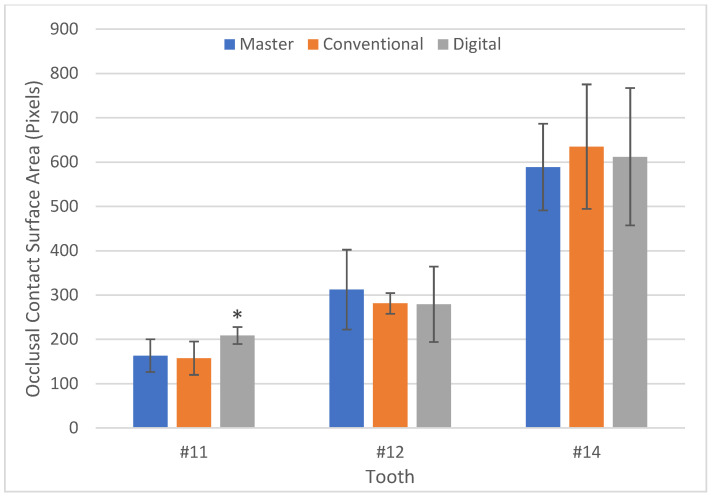
Occlusal contact surface area (pixels) post image processing. * Asterisk indicates statistical significance of *p* < 0.001.

Both the digital and conventional articulation showed mean values of the size of occlusal contacts comparable to those of the master model in terms of surface contact areas on all teeth with the exception of #11 of the digitally articulated models. The surface contact area on #11 of the digitally articulated models was significantly higher (*p* < 0.001), with a mean of 208.63 pixels versus 163.27 for the master and 157.60 for the conventionally articulated casts. There was no statistical difference between the master model and conventionally articulated casts (Group A) with regards to the surface contact area on #11 (*p* = 0.778) (Table 1, Figure 4).

**Figure 5:**
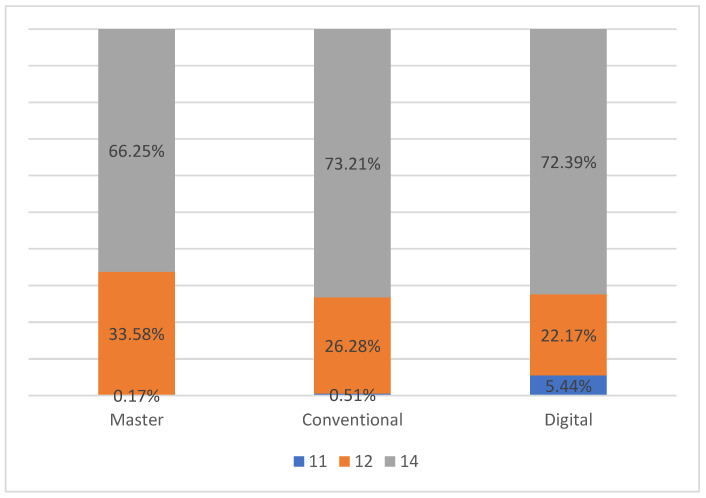
Percentage force distribution by tooth (%).

The results of the percentage of the sum of pressures on the three teeth are illustrated in Figure 5 and Table 2. The mean percentage force distributions in the master model were 0.17% on #11, 33.58% on #12 and 66.25% on 14, respectively. For the conventionally articulated casts (Group A), the mean percentage force distributions showed a similar pattern as in the master model. However, the mean percentage force distribution on #11 from the digitally mounted models (group B) was 5.44%, which was more than an order of magnitude higher when compared to 0.17% in master models and 0.51% in the conventionally mounted models (Group A).

## 4. Discussion

In the fabrication of dental restorations, the accurate articulation of dental casts is a critical component for attaining restorations in harmony with the opposing dentition. Digital technologies in the dental field have advanced to a point where restorations fabricated through a fully digital workflow have equaled or even surpassed conventional methods in terms of accuracy [27,28,29]. In a recent meta-analysis by Tabesh et al., it was concluded that intraoral scanning resulted in a superior marginal accuracy to conventional techniques for a single-unit zirconia restoration [29]. A cross-over clinical trial by Lee et al. comparing single implant restorations fabricated with conventional and digital techniques showed a comparable level of accuracy for both techniques [13]. Several studies have evaluated the accuracy of virtual interocclusal registration records for quadrant virtual casts, showing clinically acceptable results [30,31]. However, to the authors’ knowledge, none of them have looked into the occlusal accuracy of digitally mounted casts, specifically via quadrant scans and printed models.

When fabricating single unit posterior restorations on natural teeth utilizing a digital workflow, a common technique is to utilize intraoral quadrant scans of the maxillary and mandibular teeth, and a buccal bite scan for articulation. The lab utilizes these records to design and manufacture the final restoration, the fit of which is then checked and refined on 3D printed casts mounted on a simple hinge articulator. Despite the common use of this type of digital quadrant workflow for single unit restorations, little research has been done that looks at the articulation accuracy of such a system, partly due to the challenges of generating quantifiable and objective measurements when investigating occlusal pressure. Digital pressure sensors offer quantifiable data, but the measurements become highly variable and produce artifacts depending on the position of the digital pressure sensor as well as the number of occluding teeth involved.

In order to overcome these challenges and obtain meaningful data from the digital pressure sensor, this study utilized an image processing software to eliminate artifacts that could be considered as a spread of pressure rather than definitive contact areas. Dark blue readings which represented the lowest pressure areas were considered artifacts and were filtered out by adjusting the color threshold in the imaging analysis software. This allowed for a more accurate reading of the contact surface area in terms of the pixel count. This methodology of using an image processing software to refine occlusal contact data has been previously utilized by Millstein and co-authors, who utilized this software to analyze photographs of light transmission through bite registration records [19]. The force distribution values from the digital pressure sensor were converted to a percentage of the sum of pressures on the three teeth evaluated for the study. 

Based on the results of our study, we can reject our null hypothesis that there would be no difference in the accuracy of the occlusion of digitally articulated milled models and conventionally articulated stone models. Although our results showed that there was no significant difference between the master models and Group A and B in the posterior areas, in the cuspid region, Group B had a statistically larger size of the occlusal contact than in the master models. A similar trend was seen when evaluating the percentage force distribution. The percentage force distribution measured across the three tested showed that the canine had a ten-fold higher distribution percentage of 5.44% in the digitally articulated models, versus a distribution percentage of <1% in the master and conventional models. 

These findings could be attributed to multiple factors that could affect the accuracy of the articulation, including the potential difference in the accuracy of milled models versus conventional casts. The literature is limited, but two studies have shown the inferior accuracy of digitally printed models when compared to gypsum casts from polyvinylsiloxane impressions. Vögtlin and colleagues compared the accuracy of full arch digitally derived models to conventional stone casts and found stone casts to be more accurate [32]. Another study by Abduo arrived at a similar conclusion, showing an inferior accuracy of digitally derived models to gypsum casts poured from PVS impressions at the full arch level [33]. However, he concluded that digital models were actually more accurate at the tooth level.

Another possible reason for the discrepancy in the contact size could be the fact that the digital articulation was done on quadrant models versus complete arch casts for the conventional articulation. This could ostensibly result in more distribution of forces the further anteriorly you go due to the absence of additional contralateral articulating stops, thereby resulting in increased anterior contact areas in the digitally mounted quadrant models. Therefore, the conventional full arch casts appeared to reproduce an occlusal distribution pattern more similar to the master models than the digitally mounted quadrant models in the anterior area did. However, to the authors’ knowledge, there are no studies analyzing the accuracy of occlusal contacts obtained between full arch and quadrant models, and this could be an area of further study.

The limitations of this study include the aforementioned use of a full arch conventional cast versus a quadrant cast for the digital model, as well as the fact that this was an in-vitro study using a typodont on an articulator, as a result of which clinical results may differ from our findings. Another confounding factor was the use of an identical weight over both study groups, which could have affected the size of the contact areas determined through the T-scan readout.

Within the limitations of this study, the use of a digitally articulated quadrant model system may result in a loss of accuracy, in terms of occlusion, the further anteriorly the tooth to be restored is located. Thus, the clinical implications from this study are to consider the indications for using a quadrant scan in a digital workflow versus scanning more teeth in the arch to capture additional anterior or contralateral occlusal stops. The more anteriorly the tooth to be restored is located, the more capturing additional occlusal stops may improve the occlusal accuracy of the articulated models, and consequently improve the occlusal contact points of the resulting prosthesis.

## 5. Conclusions

Digital workflows in dentistry have improved the clinical experience for both patients and clinicians. However, the accuracy of these digital workflows is not always superior or equivalent to that of their conventional analogs. Much like their conventional counterparts, the multiple processes, materials, and technologies involved will contribute to an additive effect of errors, which manifests as the inaccuracy of the final restoration. Care must be taken to consider the source of these errors and work to minimize them for a more efficient and effective restorative process.

## Figures and Tables

**Table 1:** Occlusal contact surface area (pixels) post image processing.

	Master	Conventional	Digital	*p* Values
#11	163.27 ± 36.92	157.60 ± 37.70	208.63 ± 19.23	*p* < 0.001 *
#12	312.23 ± 89.99	281.20 ± 23.43	279.13 ± 84.96	*p* = 0.148
#14	588.63 ± 97.78	634.77 ± 140.52	612 ± 154.94	*p* = 0.411

* Master vs. digital: *p* < 0.001; Master vs. conventional: *p* = 0.778.

**Table 2:** Percentage force distribution by tooth (%).

	Master	Conventional	Digital
#11	0.17%	0.51%	5.44%
#12	33.58%	26.28%	22.17%
#14	66.25%	73.21%	72.39%

## Data Availability

Not applicable.
